# NF-κB activation is critical for bacterial lipoprotein tolerance-enhanced bactericidal activity in macrophages during microbial infection

**DOI:** 10.1038/srep40418

**Published:** 2017-01-12

**Authors:** Jinghua Liu, Jing Xiang, Xue Li, Siobhan Blankson, Shuqi Zhao, Junwei Cai, Yong Jiang, H. Paul Redmond, Jiang Huai Wang

**Affiliations:** 1 Key Laboratory of Functional Proteomics of Guangdong Province, Department of Pathophysiology, Southern Medical University, Guangzhou 510515, China; 2 Department of Academic Surgery, University College Cork/National University of Ireland, Cork University Hospital, Cork, Ireland

## Abstract

Tolerance to bacterial components represents an essential regulatory mechanism during bacterial infection. Bacterial lipoprotein (BLP)-induced tolerance confers protection against microbial sepsis by attenuating inflammatory responses and augmenting antimicrobial activity in innate phagocytes. It has been well-documented that BLP tolerance-attenuated proinflammatory cytokine production is associated with suppressed TLR2 signalling pathway; however, the underlying mechanism(s) involved in BLP tolerance-enhanced antimicrobial activity is unclear. Here we report that BLP-tolerised macrophages exhibited accelerated phagosome maturation and enhanced bactericidal activity upon bacterial infection, with upregulated expression of membrane-trafficking regulators and lysosomal enzymes. Notably, bacterial challenge resulted in a strong activation of NF-κB pathway in BLP-tolerised macrophages. Importantly, activation of NF-κB pathway is critical for BLP tolerance-enhanced antimicrobial activity, as deactivation of NF-κB in BLP-tolerised macrophages impaired phagosome maturation and intracellular killing of the ingested bacteria. Finally, activation of NF-κB pathway in BLP-tolerised macrophages was dependent on NOD1 and NOD2 signalling, as knocking-down NOD1 and NOD2 substantially inhibited bacteria-induced activation of NF-κB and overexpression of Rab10 and Acp5, two membrane-trafficking regulators and lysosomal enzymes contributed to BLP tolerance-enhanced bactericidal activity. These results indicate that activation of NF-κB pathway is essential for BLP tolerance-augmented antimicrobial activity in innate phagocytes and depends primarily on both NOD1 and NOD2.

A common and serious consequence of an overwhelming bacterial infection with the dysregulated systemic inflammatory response is the development of sepsis, septic shock, and their sequelae, which are the leading cause of death in intensive care units and the third cause of overall hospital mortality worldwide^1^,^2^,^3^. Despite significant achievements in our understanding of the molecular and genetic basis of sepsis and great advances in many areas of medicine over the last several decades, mortality rates of septic patients remain unacceptably high, ranging from 30% to 70%^4^,^5^,^6^. Furthermore, the incidence of sepsis and its associated economic burden continues to increase steadily by 1% every year^2^,^7^. Currently, treatment of sepsis is limited largely to antibiotics, fluid resuscitation, oxygen, and support of organ function, with no approved drugs that specifically target sepsis^1^,^8^.

The innate immune system responds rapidly through activation of pattern-recognition receptors (PRRs) upon detection of pathogen-associated molecular patterns (PAMPs), the highly conserved molecular structures of microbial pathogens and thus forms the first line of host defence against microbial infection^9^,^10^,^11^. The transmembrane Toll-like receptors (TLRs), in particular TLR4 and TLR2, are the best known PRRs and play a key role in the host defence against gram-negative and gram-positive bacterial infection by activation of TLR-mediated intracellular signal transduction pathways and initiation of both inflammatory and antimicrobial responses in innate phagocytes including polymorphonuclear neutrophils (PMNs) and monocytes/macrophages, which ultimately culminate in eliminating the invading microbial pathogens^9^,^11^,^12^,^13^. Thus, TLRs function as innate sensors of pathogen attack and alert the body to the potential of bacterial infection. However, activation of TLRs is a double-edged sword^14^. Although normally helping to eradicate microbial pathogens from a local infection, a persistent activation of TLR-mediated signalling pathways in monocytes/macrophages, characterised by the excessive release of proinflammatory cytokines and chemokines, may lead to the development of septic shock syndrome. Therefore, activation of TLR signalling pathway-induced inflammatory responses must be tightly regulated and controlled during microbial infection.

Tolerance to bacterial cell wall components represents an essential regulatory mechanism during bacterial infection^15^,^16^. The TLR4 agonist, LPS- or endotoxin-induced tolerance is a well documented phenomenon where pre-exposure to a low dose of LPS induces a transient hyporesponsive state in monocytes/macrophages with reduced production of proinflammatory cytokines, thereby conferring protection against a subsequent ‘lethal’ LPS challenge and resulting in a significant survival advantage^17^,^18^. Although the primary function of LPS tolerance is to prevent an excessive inflammatory response induced by overactivation of the TLR4 signalling pathway, acquisition of LPS tolerance has been shown to correlate with an increased incidence of secondary bacterial infection in hospitalized patients due to development of an immunosuppressive state^17^,^19^. By contrast, tolerance induced by the gram-positive bacterial cell wall component bacterial lipoprotein (BLP), a TLR2 agonist, affords protection against not only a subsequent ‘lethal’ BLP challenge but also live *Staphylococcus aureus* (*S. aureus*) and *Salmonella typhimurium* (*S. typhimurium*) infection or cecal ligation and puncture (CLP)-induced polymicrobial sepsis^20^. Notably, BLP-induced tolerance also rescues TLR4-deficient mice from gram-negative *S. typhimurium* infection with a significant survival benefit^21^. This protection, afforded by BLP tolerance, against microbial sepsis is predominantly associated with BLP-induced reprogramming in innate phagocytes characterised by hyporesponsiveness in producing proinflammatory cytokines and simultaneously, an enhanced antimicrobial activity including upregulated phagocytic receptor expression and enhanced bacterial ingestion and killing, with consequently accelerated bacterial clearance from the circulation and visceral organs^16^,^20^,^21^.

It is well described that BLP tolerance-attenuated proinflammatory cytokine production is associated with the suppressed TLR2 signalling at both the upstream and downstream pathways including reduced TLR2 and IL-1 receptor-associated kinase-1 (IRAK-1) expression, decreased myeloid differentiation factor 88 (MyD88)-IRAK immunocomplex formation, and inhibited NF-κB activation and mitogen-activated protein kinase (MAPK) phosphorylation^22^,^23^,^24^. However, the underlying signal pathways and/or molecular events responsible for BLP tolerance-augmented antimicrobial activity have not been determined. In the current study, we report that BLP-tolerised macrophages displayed accelerated phagosome maturation and enhanced bactericidal activity in response to bacterial infection. Of note, bacterial stimulation led to a strong activation of the NF-κB pathway in BLP-tolerised macrophages and this activation seems critical for BLP tolerance-augmented antimicrobial activity, as deactivation of NF-κB in BLP-tolerised macrophages impaired phagosome maturation and intracellular bacterial killing. We further show that activation of the NF-κB pathway by bacterial stimulation in BLP-tolerised macrophages appeared to be dependent on both NOD1 and NOD 2 signalling.

## Results

### BLP-tolerised macrophages show accelerated phagosome maturation and enhanced bactericidal activity in response to bacterial infection

**Figure 1:**
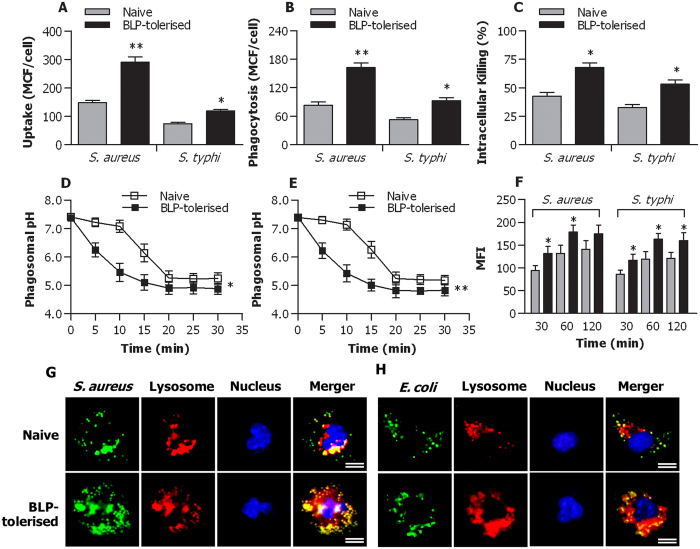
Accelerated phagosome maturation and enhanced bactericidal activity in BLP-tolerised macrophages. (**A**,**B**) Uptake (**A**) and phagocytosis (**B**) of *S. aureus* and *S. typhimurium* (*S. typhi*) at 30 min by naive and BLP-tolerised macrophages were expressed as mean channel fluorescence (MCF) per cell. (**C**) Intracellular killing of ingested *S. aureus* and *S. typhi* at 60 min by naive and BLP-tolerised macrophages. (**D**,**E**) Phagosomal pH in naive and BLP-tolerised macrophages after chased with fluorescent probe-coupled *S. aureus* (**D**) or *S. typhi* (**E**). (**F**) Phagolysosome fusion in phagosomes from naive and BLP-tolerised macrophages after chased with *S. aureus* or *S. typhi* was expressed as mean fluorescence intensity (MFI). (**G**,**H**) Naive and BLP-tolerised macrophages were loaded with LysoTraker red and further incubated with *S. aureus*-FITC (**G**) or *E. coli*-FITC (**H**). Cell nuclei were stained with DAPI. Fluorescent micrographs were taken at 60 min after incubation with either *S. aureus*-FITC or *E. coli*-FITC. Scale bar = 10 μm. All Data are mean ± SD. Data in (**A**,**B**,**D**,**E** and **F**) are from four to six independent experiments in duplicate and data in C are from five independent experiments in triplicate. *p < 0.05, **p < 0.01 compared with naive macrophages.

We first challenged naive and BLP-tolerised macrophages with gram-positive *S. aureus* and gram-negative *S. typhimurium* to examine the impact of BLP tolerisation on macrophage phagosome maturation and bactericidal activity. As shown in Fig. 1, BLP-tolerised macrophages displayed significantly enhanced uptake (*p* < 0.05, *p* < 0.01) (Fig. 1A) and phagocytosis (*p* < 0.05, *p* < 0.01) (Fig. 1B) of *S. aureus* and *S. typhimurium* compared with naive macrophages. Intracellular killing of the ingested *S. aureus* and *S. typhimurium* by BLP-tolerised macrophages was much higher than that observed in naïve macrophages (*p* < 0.05) (Fig. 1C).

A substantially accelerated phagosomal acidification after ingestion of *S. aureus* (*p* < 0.05) (Fig. 1D) or *S. typhimurium* (*p* < 0.01) (Fig. 1E) was observed in BLP-tolerised macrophages compared with naive macrophages. Consistent with the accelerated phagosomal acidification, BLP-tolerised macrophages exhibited significantly increased phagolysosome fusion at 30, 60, and 120 min after ingestion of *S. aureus* or *S. typhimurium* (*p* < 0.05 versus naive macrophages) (Fig. 1F) as determined in a cell-free organelle system.

We further loaded naive and BLP-tolerised macrophages with LysoTracker red that selectively labels late endosomes/lysosomes and monitored the maturation of phagosomes that have ingested *S. aureus*-FITC or *E. coli*-FITC by examining their ability to colocalise with LysoTracker red over time. A markedly increased colocalisation of either *S. aureus*-FITC (Fig. 1G) or *E. coli*-FITC (Fig. 1H) with LysoTraker red was observed in BLP-tolerised macrophages compared with naive macrophages. Collectively, these results indicate that in comparison with naive macrophages, BLP-tolerised macrophages are characterised with accelerated phagosome maturation and enhanced bactericidal activity in response to microbial infection.

### BLP-tolerised macrophages display upregulated expression of membrane-trafficking regulators and lysosomal enzymes after bacterial infection

Upregulation and activation of membrane-trafficking regulators and lysosomal enzymes in innate phagocytes such as macrophages during the process of bacterial phagocytosis are critical events for subsequent phagosome/lysosome fusion and an efficient killing of the internalized pathogens within the phagocyte^25^,^26^,^27^. We next compared the gene expression profile of phagosome maturation-associated membrane-trafficking regulators and lysosomal enzymes between naive and BLP-tolerised bone marrow-derived macrophages (BMMs). Among the 56 genes of membrane-trafficking regulators and lysosomal enzymes analysed, 7 genes including Acp2, Acp5, Rab10, Rab20, Rabgef1, Camp, and Ctsc were upregulated in BLP-tolerised BMMs compared with naive BMMs (Supplementary Table 1).

**Figure 2:**
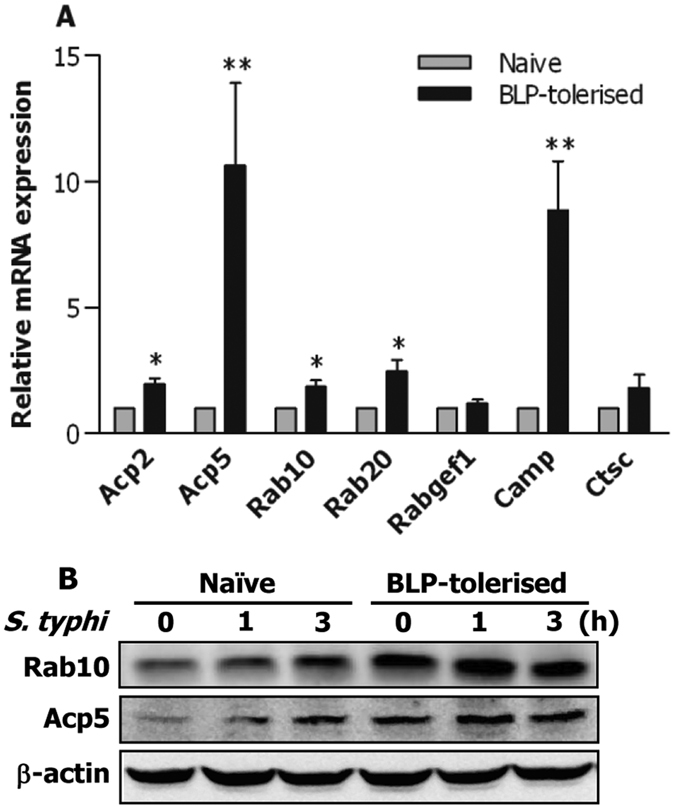
Up-regulated expression of membrane-trafficking regulators and lysosomal enzymes in BLP-tolerised macrophages. (**A**) Expression of Acp2, Acp5, Rab10, Rab 20, Rabgef1, Camp, and Ctsc mRNA in naive and BLP-tolerised BMMs was assessed by quantitative real-time RT-PCR. Data are mean ± SD from at least three independent experiments and each experiment was conducted in duplicate. *p < 0.05, **p < 0.01 compared with naive BMMs. (**B**) Expression of Rab10 and Acp5 protein in naive and BLP-tolerised BMMs at the indicated time points after *S. typhimurium* (*S. typhi*) stimulation was assessed by Western blot analysis. Results shown represent one experiment from a total of three separate experiments.

To validate the above findings, we further assessed mRNA and protein expressing levels of these upregulated genes and compared them between naive and BLP-tolerised BMMs. Significantly increased mRNA levels of Acp2, Acp5, Rab10, Rab20, and Camp were observed in BLP-tolerised BMMs (*p* < 0.05, *p* < 0.01 versus naive BMMs) (Fig. 2A). Although Rabgef1 and Ctsc mRNA levels in BLP-tolerised BMMs were higher than those in naive BMMs, they did not reach statistical significances (Fig. 2A). Western blot analysis further revealed that BLP-tolerised BMMs displayed upregulated protein expression of both Rab10 and Acp5 over naive BMMs before bacterial challenge (Fig. 2B), while *S. typhimurium* infection led to further increases in Rab10 and Acp5 protein expression in BLP-tolerised BMMs compared with naive BMMs (Fig. 2B). These results indicate that BLP-tolerised macrophages are featured with upregulation of membrane-trafficking regulators and lysosomal enzymes.

**Figure 3:**
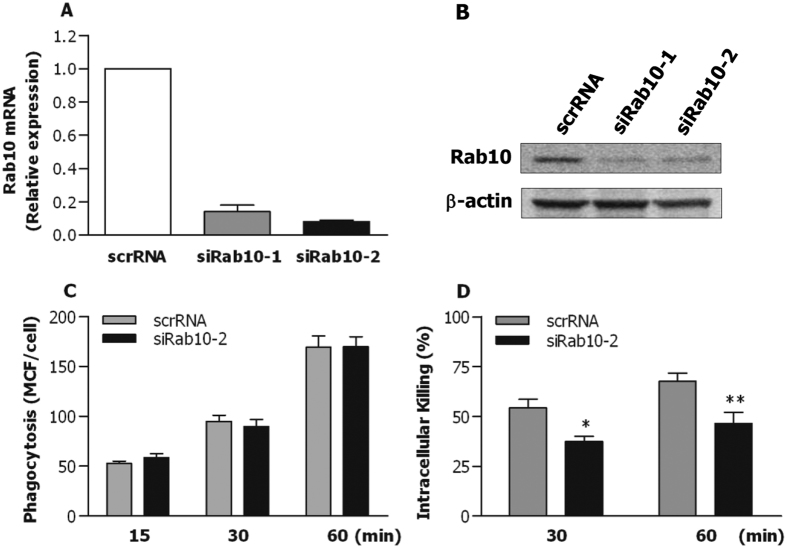
Silencing Rab10 diminishes BLP-tolerised macrophage-induced intracellular bacterial killing. (**A,B**) Murine BMMs were transfected with two Rab10 specific siRNA, siRab10-1 and siRab10-2, or its scrambled siRNA (scrRNA). Expression of Rab10 mRNA (**A**) and protein (**B**) was assessed by quantitative real-time RT-PCR and Western blot analysis 24 hrs after transfection. (**C,D**) Phagocytosis of *S. typhimurium* (**C**) and intracellular killing of the ingested *S. typhimurium* (**D**) by BLP-tolerised BMMs transfected with either scrRNA or siRab10-2. Data are mean ± SD from four to six independent experiments in duplicate or triplicate. *p < 0.05, **p < 0.01 compared with BLP-tolerised BMMs transfected with scrRNA.

We next examined whether upregulated expression of membrane-trafficking regulators and lysosomal enzymes are required for the enhanced bactericidal activity seen in BLP-tolerised macrophages by silencing Rab10 expression. Transfection of murine BMMs with Rab10 specific small interfering RNA (siRNA), siRab10-1 and siRab10-2, efficiently knocked down Rab10 expression at both the mRNA (Fig. 3A) and the protein (Fig. 3B) levels compared with BMMs transfected with scrambled siRNA (scrRNA). Knockdown of Rab10 with siRab10-2 did not affect BLP-tolerised BMMs to ingest *S. typhimurium* (Fig. 3C), but markedly impaired intracellular killing of the ingested *S. typhimurium* by BLP-tolerised BMMs (*p* < 0.05, *p* < 0.01 versus BLP-tolerised BMMs transfected with scrambled siRNA) (Fig. 3D), suggesting that upregulated expression of membrane-trafficking regulators and lysosomal enzymes contributes to an enhanced bactericidal activity in BLP-tolerised macrophages.

### Bacterial stimulation leads to activation of the NF-κB pathway in BLP-tolerised macrophages

**Figure 4:**
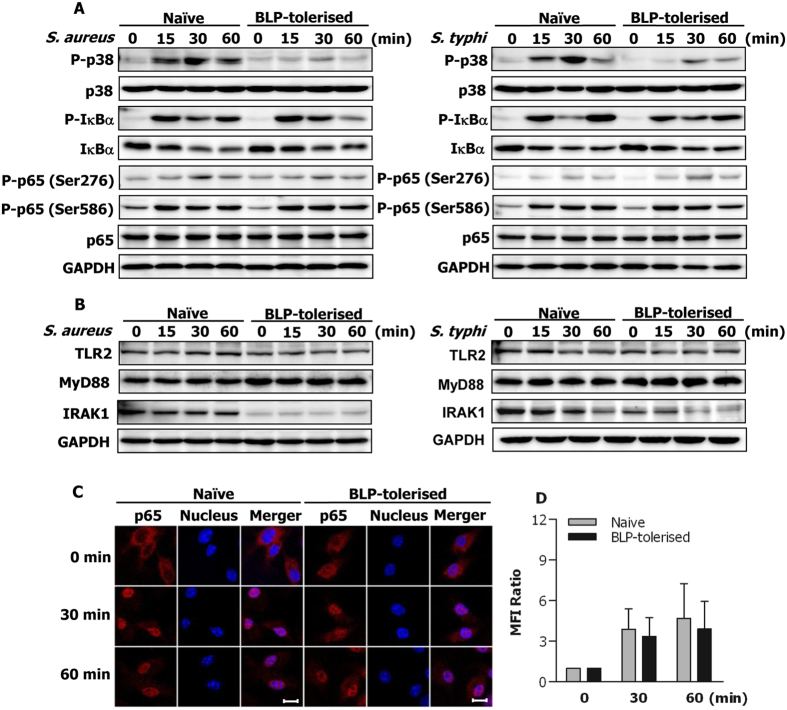
Bacterial stimulation-induced activation of the NF-κB pathway in BLP-tolerised macrophages. Naive and BLP-tolerised BMMs were stimulated with *S. aureus* or *S. typhimurium* (*S. typhi*) for the indicated time periods. (**A**,**B**) Cytoplasmic proteins were extracted and subjected to immunoblotting for detection of total and phosphorylated p38 (P-p38), total and phosphorylated IκBα (P-IκBα), total and phosphorylated p65 (P-p65), TLR2, MyD88, and IRAK-1. Results shown represent one experiment from a total of three separate experiments. (**C**,**D**) Confocal images were taken at the indicated time points after naive and BLP-tolerised BMMs were subjected to *S. typhimurium* infection by immunofluorescent staining with the anti-p65 Ab and Alexa Flour 594-conjugated secondary Ab (**C**), and nuclear translocation of p65 was quantitatively analysed and expressed as mean fluorescence intensity (MFI) ratio (**D**). Results shown represent one experiment from a total of three separate experiments. Cell nuclei were stained with DAPI. Scale bar = 10 μm.

Our previous work has shown that BLP-induced self-tolerance and cross-tolerance to LPS are associated with the suppressed TLR2 signalling at both the upstream and downstream pathways^22^,^23^,^24^. We therefore attempted to examine the influence of bacterial infection on TLR2-mediated intracellular signal transduction pathways in naive and BLP-tolerised macrophages. As shown in Fig. 4A, stimulation of naive BMMs with *S. aureus* or *S. typhimurium* resulted in substantial activations in the downstream pathways of TLR2 signalling including phosphorylation of MAPK p38, the inhibitor of κBα (IκBα), and NF-κB p65, whereas an almost complete suppression of MAPK p38 phosphorylation was observed in BLP-tolerised BMMs after stimulation with *S. aureus* or *S. typhimurium*. Surprisingly, BLP-tolerised BMMs showed strong activation of the NF-κB pathway with substantially enhanced phosphorylation of IκBα at Ser32 and NF-κB p65 at Ser586 in response to either *S. aureus* or *S. typhimurium* stimulation (Fig. 4A), which is largely different from our previous findings^22^,^23^ where BLP-tolerised monocytes/macrophages exhibited suppressed IκBα phosphorylation and reduced NF-κB-DNA binding activity in response to a second BLP or LPS stimulation. Expression of the upstream pathways of TLR2 signalling including TLR2, MyD88, and IRAK-1 in BLP-tolerised BMMs after stimulation with *S. aureus* or *S. typhimurium* (Fig. 4B) was similar to those seen previously in BLP-tolerised monocytes/macrophages after stimulation with BLP or LPS^22^,^23^,^24^.

To further confirm bacterial stimulation-induced NF-κB activation in BLP-tolerised macrophages, we monitored the nuclear translocation of NF-κB p65 upon *S. typhimurium* infection in both naive and BLP-tolerised macrophages. Before bacterial challenge, almost all of the detected p65 staining was located in the cytoplasm of naive BMMs, whereas some positive staining for p65 was also observed in the nucleus of BLP-tolerised BMMs (Fig. 4C) (Supplementary Fig. S1). In response to *S. typhimurium* infection, substantially enhanced translocation of p65 from the cytoplasm into the nucleus was evident not only in naive macrophages but also in BLP-tolerised BMMs (Fig. 4C,D) (Supplementary Fig. S1). Together, these results indicate that bacterial stimulation, in contrast to a second BLP or LPS stimulation, activates the NF-κB pathway in BLP-tolerised macrophages.

### Activation of the NF-κB pathway is responsible for accelerated phagosome maturation and enhanced bactericidal activity in BLP-tolerised macrophages

**Figure 5:**
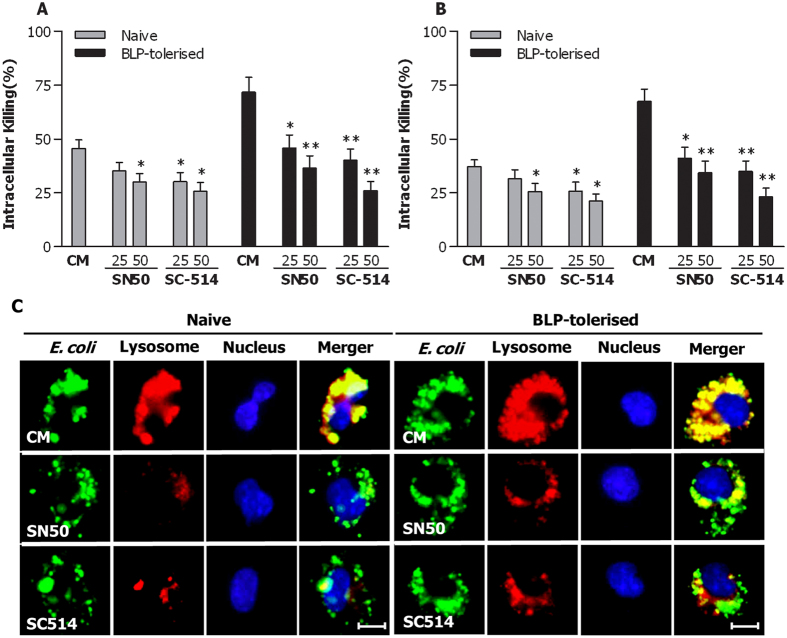
Inhibition of NF-κB activation impairs phagosome maturation and bactericidal activity in BLP-tolerised macrophages. (**A**,**B**) Naive and BLP-tolerised macrophages pretreated with two NF-κB inhibitors SN50 (25, 50 μg/ml), SC-514 (25, 50 nM) or culture medium (CM) for 60 min were stimulated with *S. aureus* or *S. typhimurium* to assess intracellular killing of the ingested *S. aureus* (**A**) and *S. typhimurium* (**B**). Data are mean ± SD from five independent experiments in triplicate. *p < 0.05, **p < 0.01 compared with naive or BLP-tolerised macrophages pretreated with CM. (**C**) Naive and BLP-tolerised macrophages pretreated with SN50 (50 μg/ml) or SC514 (50 nM) were loaded with LysoTraker red and further incubated with *E. coli*-FITC. Cell nuclei were stained with DAPI. Fluorescent micrographs were taken at 60 min after incubation with *E. coli*-FITC. Scale bar = 10 μm.

To ascertain whether bacteria-induced activation of the NF-κB pathway is associated with accelerated phagosome maturation and enhanced bactericidal activity characterised in BLP-tolerised macrophages, we abrogated the NF-κB pathway using two specific NF-κB inhibitors, SN50 and SC-514. Pretreatment with either SN50 or SC-514 significantly reduced intracellular killing of *S. aureus* (Fig. 5A) and *S. typhimurium* (Fig. 5B) in naive macrophages (*p* < 0.05 versus naive macrophages pretreated with culture medium); however, a much stronger impairment in bactericidal activity with substantially diminished intracellular killing of both *S. aureus* (Fig. 5A) and *S. typhimurium* (Fig. 5B) was observed in SN50-pretreated or SC-514-pretreated, BLP-tolerised macrophages (*p* < 0.05, *p* < 0.01 versus BLP-tolerised macrophages pretreated with culture medium). Furthermore, inhibition of NF-κB activation with either SN50 or SC-514 markedly delayed phagolysosome fusion in BLP-tolerised macrophages after ingestion of *E. coli* compared with BLP-tolerised macrophages pretreated with culture medium (Fig. 5C).

**Figure 6:**
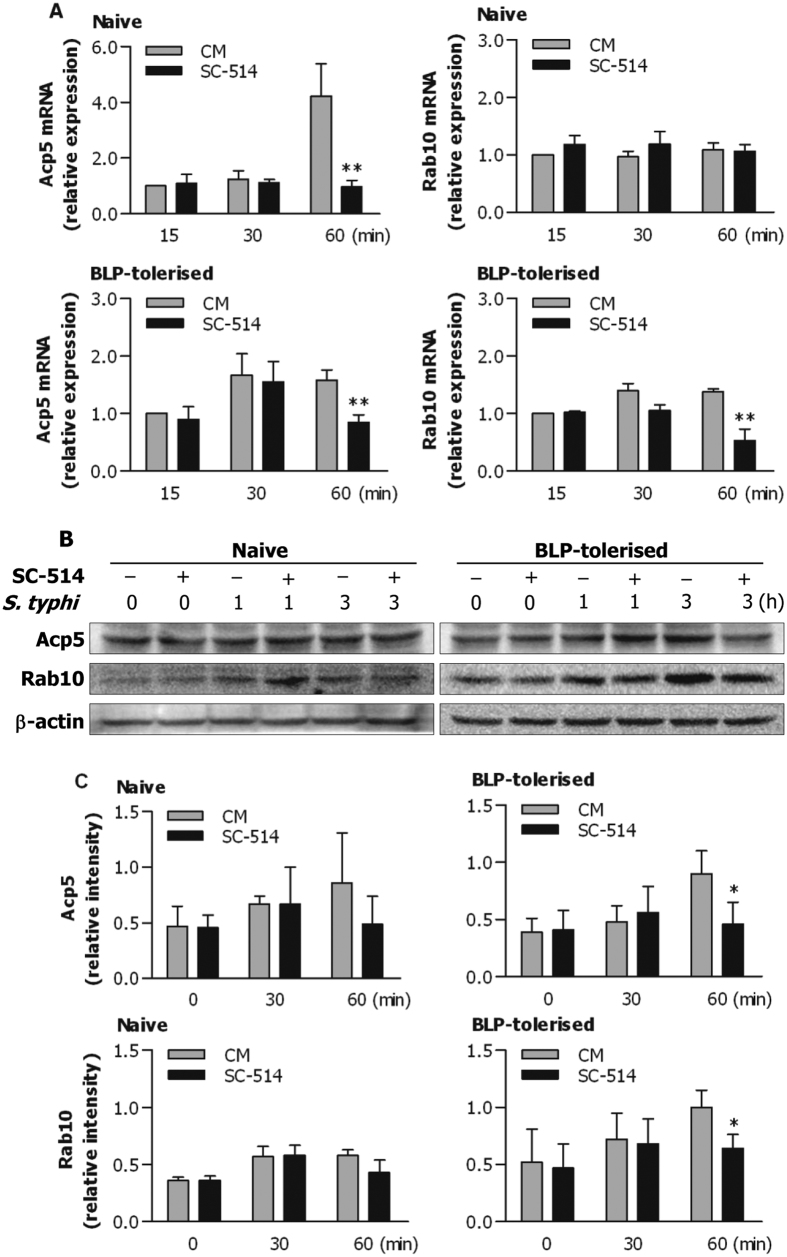
Inhibition of NF-κB activation attenuates Acp5 and Rab10 expression in BLP-tolerised macrophages. Naive and BLP-tolerised BMMs pretreated with the NF-κB inhibitors SC-514 (50 nM) or culture medium (CM) for 60 min were stimulated with *S. typhimurium* (*S. typhi*) for the indicated time periods. (**A**) Expression of Acp5 and Rab10 mRNA was assessed by quantitative real-time RT-PCR. Data are mean ± SD from four to five independent experiments and each experiment was conducted in duplicate. **p < 0.01 compared with naive or BLP-tolerised BMMs pretreated with CM. (**B**) Expression of Acp5 and Rab10 protein was assessed by Western blot analysis. Results shown represent one experiment from a total of three separate experiments. (**C**) The relative intensity of Acp5 and Rab10 was analysed by densitometry. *p < 0.05 compared with BLP-tolerised BMMs pretreated with CM.

As upregulation of membrane-trafficking regulators and lysosomal enzymes contributes to an enhanced bactericidal activity in BLP-tolerised macrophages, we next examined whether inhibition of NF-κB activation affects Acp5 and Rab10 expression at the mRNA and protein levels. Pretreatment of naive BMMs with SC-514 significantly attenuated mRNA expression of Acp5, but not Rab10, at 60 min after *S. typhimurium* infection (*p* < 0.01 versus naive BMMs pretreated with culture medium), whereas SC-514 pretreatment substantially abrogated *S. typhimurium*-induced mRNA expression of both Acp5 and Rab10 in BLP-tolerised BMMs (*p* < 0.01 versus BLP-tolerised BMMs pretreated with culture medium) (Fig. 6A). Notably, pretreatment with SC-514 significantly attenuated *S. typhimurium*-enhanced protein expression of Acp5 and Rab10 in BLP-tolerised BMMs (*p* < 0.05 versus BLP-tolerised BMMs pretreated with culture medium) (Fig. 6B,C), but not in naive BMMs (Fig. 6B,C). Collectively, these results indicate that activation of the NF-κB pathway by bacterial infection is the prerequisite for the observed augmentation of bactericidal activity in BLP-tolerised macrophages, partly via upregulation of membrane-trafficking regulators and lysosomal enzymes.

### Bacteria-activated NF-κB pathway in BLP-tolerised macrophages is dependent on NOD1 and NOD2

**Figure 7:**
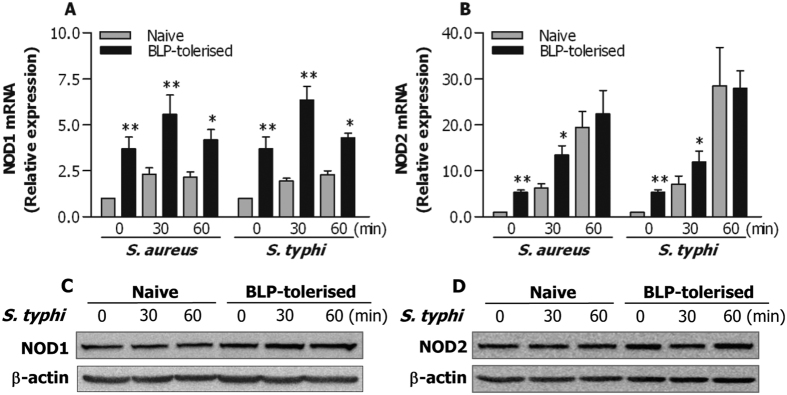
Enhanced mRNA expression of NOD1 and NOD2 in BLP-tolerised macrophages in response to bacterial stimulation. (**A**,**B**) Naive and BLP-tolerised BMMs were stimulated with *S. aureus* or *S. typhimurium* for the indicated time periods. Expression of NOD1 and NOD2 mRNA was assessed by quantitative real-time RT-PCR. Data are mean ± SD from four to five independent experiments and each experiment was conducted in duplicate. *p < 0.05, **p < 0.01 compared with naive BMMs. (**C**,**D**) Expression of NOD1 and NOD2 protein in naive and BLP-tolerised BMMs after stimulation with *S. typhimurium* (*S. typhi*) for the indicated time periods was assessed by Western blot analysis. Results shown represent one experiment from a total of three separate experiments.

NOD1 and NOD2 are two members of the nucleotide binding and oligomerization domain (NOD)-like receptor (NLR) family, located in the cytoplasm, and belong to the intracellular PRRs^28^,^29^,^30^. Engagement of NOD1 and/or NOD2 with bacterial ligands predominantly leads to activation of their downstream NF-κB pathway^31^,^32^. Therefore, we asked whether NOD1 and NOD2 are involved in bacteria-activated NF-κB pathway in BLP-tolerised macrophages, thus facilitating an enhanced bactericidal activity. We first assessed NOD1 and NOD2 expression in naive and BLP-tolerised macrophages before and after bacterial challenge. As shown in Fig. 7A,B, BLP-tolerised BMMs exhibited significantly higher mRNA expression of both NOD1 and NOD2 than naive BMMs before bacterial challenge (*p* < 0.01). Substantially increases in mRNA levels of NOD1 (Fig. 7A) and NOD2 (Fig. 7B) were seen in both naive and BLP-tolerised BMMs in response to *S. aureus* or *S. typhimurium* infection; however, much higher mRNA expression of NOD1 and NOD2 was seen in BLP-tolerised BMMs compared with naive BMMs (*p* < 0.05, *p* < 0.01) (Fig. 7A,B). We further assessed protein expression of NOD1 and NOD2 in response to *S. typhimurium* infection in naive and BLP-tolerised BMMs. Surprisingly, in contrast to the enhanced mRNA expression, no obvious increases in protein expression of both NOD1 and NOD2 were observed in BLP-tolerised BMMs after *S. typhimurium* stimulation compared with naive BMMs (Fig. 7C,D).

**Figure 8:**
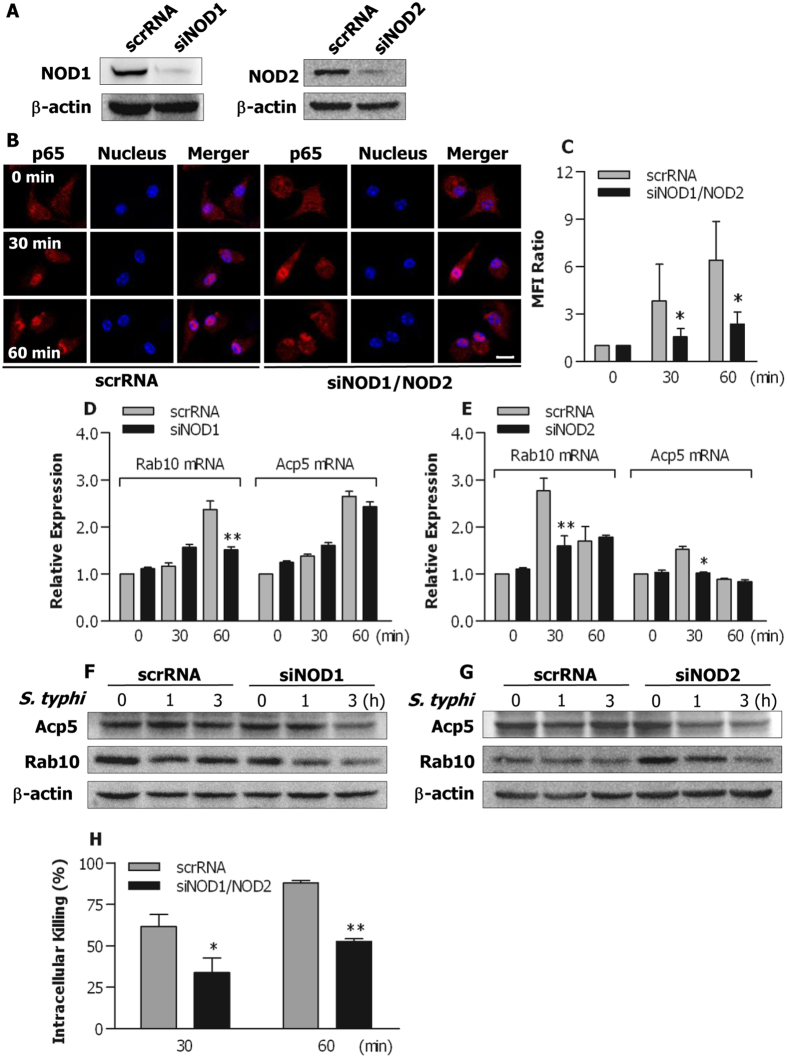
Both NOD1 and NOD2 are involved in bacteria-stimulated activation of the NF-κB pathway in BLP-tolerised macrophages. (**A**) Murine BMMs were transfected with the NOD1 specific siRNA (siNOD1), NOD2 specific siRNA (siNOD2), or their scrambled siRNA (scrRNA). Expression of NOD1 and NOD2 protein was assessed by Western blot analysis 24 hrs after transfection. (**B**,**C**) BLP-tolerised BMMs transfected with siNOD1/NOD2 or scrRNA were stimulated with *S. typhimurium* for the indicated time periods. Confocal images were taken after cells were stained with the anti-p65 Ab and Alexa Flour 594-conjugated secondary Ab (**B**), and nuclear translocation of p65 was quantitatively analysed and expressed as mean fluorescence intensity (MFI) ratio (**C**). Results shown represent one experiment from a total of three separate experiments. Cell nuclei were stained with DAPI. Scale bar = 10 μm. (**D**–**G**) BLP-tolerised BMMs transfected with siNOD1, siNOD2, or their scrRNA were stimulated with *S. typhimurium* (*S. typhi*) for the indicated time periods. Expression of Acp5 and Rab10 mRNA (**D**,**E**) was assessed by quantitative real-time RT-PCR. Expression of Acp5 and Rab10 protein (**F**,**G**) was assessed by Western blot analysis. Results shown represent one experiment from a total of three separate experiments. (**H**) Intracellular killing of the ingested *S. typhimurium* by BLP-tolerised BMMs transfected with either siNOD1/NOD2 or scrRNA. All data are mean ± SD from four to six independent experiments in duplicate or triplicate. *p < 0.05, **p < 0.01 compared with BLP-tolerised BMMs transfected with scrRNA.

We next examined whether knockdown of NOD1 and NOD2 with their specific siRNA affects bacteria-stimulated NF-κB activation in BLP-tolerised macrophages. Transfection of murine BMMs with the NOD1 specific siRNA (siNOD1) or NOD2 specific siRNA (siNOD2) efficiently knocked down NOD1 and NOD2 protein expression compared with BMMs transfected with their scrRNA) (Fig. 8A). Of note, markedly increased nuclear translocation of cytoplasmic NF-κB p65 was seen in BLP-tolerised BMMs transfected with scrRNA at 30 and 60 min after *S. typhimurium* infection, whereas doubly knocking-down NOD1 and NOD2 substantially reduced *S. typhimurium*-stimulated translocation of p65 from the cytoplasm into the nucleus in BLP-tolerised BMMs (p < 0.05 versus BLP-tolerised BMMs transfected with scrRNA) (Fig. 8B,C) (Supplementary Fig. S2), suggesting that blocking NOD1 and NOD2 inhibits bacteria-induced NF-κB activation in BLP-tolerised macrophages.

We further determined whether knockdown of either NOD1 or NOD2 influences membrane-trafficking regulator and lysosomal enzyme Acp5 and Rab10 expression in BLP-tolerised macrophages. *S. typhimurium* infection resulted in markedly enhanced mRNA expression of Rab10 and Acp5 in BLP-tolerised BMMs (Fig. 8D,E). Knocking-down NOD1 in BLP-tolerised BMMs strongly inhibited *S. typhimurium*-induced upregulation of Rab10 mRNA, but not of Acp5 mRNA (*p* < 0.01 versus BLP-tolerised BMMs transfected with scrRNA) (Fig. 8D), whereas knocking-down NOD2 in BLP-tolerised macrophages markedly suppressed *S. typhimurium*-induced mRNA expression of both Acp5 and Rab10 (*p* < 0.05, *p* < 0.01 versus BLP-tolerised BMMs transfected with scrRNA) (Fig. 8E). Moreover, Knocking-down either NOD1 (Fig. 8F) or NOD2 (Fig. 8G) resulted in a substantial attenuation in protein expression of both Acp5 and Rab10. Critically, knockdown of both NOD1 and NOD2 in BLP-tolerised BMMs strongly impaired bactericidal activity with significantly diminished intracellular killing of the ingested *S. typhimurium* (*p* < 0.05, *p* < 0.01 versus BLP-tolerised BMMs transfected with scrRNA) (Fig. 8H). These results indicate that both NOD1 and NOD2 contribute primarily to bacteria-stimulated activation of the NF-κB pathway, upregulation of membrane-trafficking regulators and lysosomal enzymes, and subsequently augmented bactericidal activity seen in BLP-tolerised macrophages.

## Discussion

BLP tolerance-afforded protection against microbial sepsis is closely associated with BLP-induced reprogramming in innate phagocytes characterised by hyporesponsiveness in producing proinflammatory cytokines and simultaneously, an augmented antimicrobial activity^16^,^20^,^21^. Although the signal transduction pathway underlying BLP tolerance-attenuated inflammatory responses has been well described^22^,^23^,^24^, mechanism(s) involved in BLP tolerance-enhanced antimicrobial activity in phagocytes is undetermined. In the current study, we demonstrated that BLP-tolerised macrophages exhibited accelerated phagosome maturation and enhanced bactericidal activity in response to bacterial infection. Notably, stimulation of BLP-tolerised macrophages with bacteria led to a strong activation of the NF-κB pathway. Importantly, activation of the NF-κB pathway appeared to be a prerequisite for BLP tolerance-induced augmentation of antimicrobial activity and predominantly dependent on both NOD1 and NOD2.

We first examined whether isolated murine macrophages after induction of BLP tolerance developed an enhanced antimicrobial activity in response to bacterial infection. Significantly enhanced uptake and phagocytosis of both gram-positive *S. aureus* and gram-negative *S. typhimurium* were observed in BLP-tolerised macrophages compared with naive macrophages. The antimicrobial response of innate immunity is initiated by the receptor-associated recognition of the invading microbial pathogens and subsequently, these pathogens are engulfed by innate phagocytes and killed within the phagocyte through a process of phagosome/lysosome fusion^26^,^33^,^34^. Thus, phagosome maturation after ingestion of microbial pathogens, characterised by phagosomal acidification and phagolysosome fusion, is a critical step in the killing and degradation of the internalised pathogens. We next asked whether induction of BLP tolerance in macrophages affects phagosome maturation upon bacterial ingestion, thus facilitating intracellular killing of the internalised pathogens. By measuring both phagosomal acidification and phagolysosome fusion, we confirmed that BLP-tolerised macrophages were characterised with accelerated phagosome maturation. Consequently, substantially increased intracellular killing of the ingested *S. aureus* and *S. typhimurium* was evident in BLP-tolerised macrophages compared with naive macrophages.

Activation and/or upregulation of membrane-trafficking regulators and lysosomal enzymes in innate phagocytes during the process of bacterial phagocytosis are critical events for phagosome formation, phagosome/lysosome fusion, and subsequent efficient killing of the internalized pathogens^25^,^26^,^27^. For instance, perturbation of Rab5 or Rab7 activation impairs late phagosome and phagolysosome formation^35^, and blocks phagosomal acidification and phagosome/lysosome fusion^36^,^37^. Moreover, it has been shown that the lysosome-associated membrane protein (LAMP) is essential not only for recruitment of Rab7 during phagolysosome fusion but also for the acquisition of bactericidal activity in the phagosome^38^,^39^. Therefore, we examined whether an enhanced bactericidal activity observed in BLP-tolerised macrophages is linked to the upregulated membrane-trafficking regulators and lysosomal enzymes. RT2 profiler PCR arrays and quantitative real-time RT-PCR revealed that induction of BLP tolerance in murine BMMs upregulated gene expression of membrane-trafficking regulators and lysosomal enzymes. Western blot analysis demonstrated that expression of Rab10 and Acp5 proteins was further enhanced in response to bacterial stimulation in BLP-tolerised BMMs. Critically, knockdown of Rab10 dramatically impaired BLP-tolerised BMM-associated intracellular killing of the ingested microbial pathogens, indicating the involvement of membrane-trafficking regulators and lysosomal enzymes in BLP tolerance-enhanced bactericidal activity.

Our previous work has demonstrated that BLP-induced tolerance in monocytes/macrophages is associated with the suppressed TLR2 signalling at both the upstream and downstream pathways, with diminished expression of TLR2 and IRAK-1, reduced formation of MyD88-IRAK immunocomplex, and inhibited activation of both NF-κB and MAPKs in response to a second BLP or LPS stimulation^22^,^23^,^24^. However, it is unclear the impact of bacterial infection on TLR2-mediated signal transduction pathways in BLP-tolerised macrophages. We found that expression of TLR2, MyD88, and IRAK-1, the upstream components of TLR2 signalling, and phosphorylation of MAPK p38, one of the downstream pathways of TLR2 signalling, in BLP-tolerised BMMs after stimulation with *S. aureus* or *S typhimurium* were similar to those seen in BLP-tolerised monocytes/macrophages after stimulation with BLP or LPS^22^,^23^,^24^. By contrast, phosphorylation of IκBα and NF-κB p65, and translocation of p65 from the cytoplasm into the nucleus in BLP-tolerised BMMs were substantially enhanced in response to *S. aureus* or *S. typhimurium* stimulation, demonstrating that bacterial stimulation activates the NF-κB pathway, a more predominant downstream pathway of TLR2 signalling, in BLP-tolerised macrophages. NF-κB is a central regulator for both innate and adaptive immune functions^40^,^41^, and NF-κB activation has been shown to promote the antimicrobial response by facilitating bacterial phagocytosis and killing in macrophages^42^,^43^. Therefore, we hypothesised that activation of the NF-κB pathway by bacterial infection is responsible for an augmented antimicrobial activity characterised in BLP-tolerised macrophages. To test this, we abrogated the NF-κB pathway with its specific inhibitors. As expected, ablation of NF-κB activation substantially blocked phagolysosome fusion and impaired intracellular killing of both the ingested *S. aureus* and *S. typhimurium* in BLP-tolerised macrophages. Moreover, inhibition of NF-κB in BLP-tolerised BMMs strongly attenuated *S. typhimurium*-induced overexpression of Acp5 and Rab10, two membrane-trafficking regulators and lysosomal enzymes linked to BLP tolerance-enhanced bactericidal activity.

The remaining question is that by which signal pathway and/or molecular event bacterial stimulation activates the NF-κB pathway in BLP-tolerised macrophages. The membrane-bound TLRs and the cytoplasmic NLRs are two major classes of PRRs that are primarily responsible for recognition of PAMPs of bacterial origin^11^,^13^,^28^,^44^. Similar to TLR signalling, upon recognition of bacterial ligands both NOD1 and NOD2 initiate the targeted gene transcription by activating the NF-κB transcription factor signalling pathway via recruitment of the adaptor proteins RICK and caspase recruitment domain 9 (CARD9)^31^,^32^,^45^. Therefore, we speculated that bacteria-stimulated activation of the NF-κB pathway in BLP-tolerised macrophages is via NOD1 and/or NOD2 signalling. Although we found upregulated mRNA expression, we did not detect enhanced protein levels of NOD1 and NOD2 in BLP-tolerised BMMs after bacterial infection. However, knockdown of both NOD1 and NOD2 impaired bacteria-induced activation of the NF-κB pathway in BLP-tolerised BMMs, as evidenced by significantly reduced nuclear translocation of p65 in response to bacterial infection. Furthermore, silencing NOD1 or NOD2 in BLP-tolerised BMMs markedly suppressed bacteria-stimulated upregulation of membrane-trafficking regulators and lysosomal enzymes Acp5 and Rab10. Finally, knockdown of both NOD1 and NOD2 substantially diminished intracellular killing of the ingested *S. typhimurium* by BLP-tolerised BMMs. These results highlight the involvement of both NOD1 and NOD2 signalling in bacteria-induced activation of the NF-κB pathway and subsequently enhanced bactericidal activity in BLP-tolerised macrophages.

In conclusion, we demonstrate that in response to bacterial infection BLP-tolerised macrophages are characterised with accelerated phagosome maturation and enhanced bactericidal activity. Furthermore, bacterial stimulation leads to a strong activation of the NF-κB pathway in BLP-tolerised macrophages via both NOD1 and NOD2 signalling. More importantly, Bacteria-stimulated NF-κB activation is critical for BLP tolerance-induced augmentation of antimicrobial activity. These findings suggest that modulation of the TLR2 signalling in innate phagocytes to augment the bactericidal activity may represent a potential therapeutic strategy during microbial infection.

## Methods

### Reagents and antibodies

The TLR2 agonist BLP, a synthetic bacterial lipopeptide (Pam_3_Cys-Ser-Lys_4_-OH), was purchased from EMC Microcollections (Tubingen, Germany). Abs that recognize TLR2, MyD88, and IRAK1 were purchased from Santa Cruz Biotechnology (Santa Cruz, CA, USA) and Abcam (Cambridge, MA, USA), respectively. Abs that recognize p38, phospho-p38, IκBα, phosphor-IκBα at Ser32, p65, phosphor-p65 at Ser276 or at Ser586, Acp5, Rab10, NOD1, and NOD2 were purchased from Santa Cruz Biotechnology and Cell Signalling Technology (Beverly, MA, USA), respectively. siRNA specifically targeting NOD1, NOD2, Rab10, and their scrRNA were obtained from GenePharma (Shanghai, China). The NF-κB inhibitors SN50 and SC-514 were purchased from Merck Millipore (Billerica, MA, USA). All culture medium and reagents used for cell cultures were purchased from Invitrogen Life Technologies (Paisley, Scotland, U.K.). All other chemicals, unless indicated, were from Sigma-Aldrich (St. Louis, MO, USA).

### Mice, murine macrophage isolation and cultures

Pyrogen-free, 8- to 10-wk-old C57BL/6 mice were purchased from Harlan (Oxon, U.K.). Mice were housed in barrier cages under controlled environmental conditions (12/12 hrs light/dark cycle, 55 ± 5% humidity, 23 °C) in the University Biological Services Unit, University College Cork. All animal procedures were performed in the University Biological Services Unit under a license from the Department of Health (Republic of Ireland) and with ethical approval granted from the University College Cork Ethics Committee. The methods applied in this study were carried out in accordance with the approved guidelines.

Peritoneal macrophages were collected from C57BL/6 mice by peritoneal lavage and incubated with DMEM containing 10% heat-inactivated FCS for 90 min to remove non-adherent cells, as described previously^46^. BMMs were isolated from the femurs and tibias of C57BL/6 mice and cultured in DMEM containing 20% heat-inactivated FCS, penicillin (100 units/ml), streptomycin sulfate (100 μg/ml), and supplemented with 10 ng/ml of recombinant mouse macrophage-CSF (R&D Systems, Minneapolis, MN, USA) for 7 days at 37 °C in a humidified 5% CO2 atmosphere, as described previously^46^. The purity of both peritoneal macrophages and BMMs was >95%, as confirmed by FAC Scan analysis of the positive F4/80 Ag staining with a rat anti-mouse F4/80 Ab (Serotec, Oxford, U.K.).

### Induction of BLP tolerance

For induction of BLP tolerance, isolated peritoneal macrophages or BMMs were pretreated with culture medium (naive) or BLP at 100 ng/ml (BLP-tolerised) for 24 hrs before bacterial challenge, as described previously^22^,^23^,^24^.

### Bacteria and bacterial uptake, ingestion, and killing

Gram-positive *S. aureus* and gram-negative *S. typhimurium* were obtained from American Type Culture Collection (ATCC, Manassas, VA, USA) and the National University of Ireland Culture Collection, respectively. Bacteria were cultured at 37 °C in trypticase soy broth (Merck, Darmstadt, Germany), harvested at the mid-logarithmic growth phase, washed twice, and resuspended in PBS for *in vitro* use. The concentration of resuspended bacteria was determined and adjusted spectrophotometrically at 550 nm.

Bacterial uptake, phagocytosis, and intracellular bacterial killing were determined as described previously^21^,^47^. Briefly, *S. aureus* and *S. typhimurium* were heat-killed at 95 °C for 20 min and labelled with 0.1% FITC (Sigma-Aldrich). Isolated peritoneal macrophages or BMMs were incubated with heat-killed, FITC-labelled *S. aureus* or *S. typhimurium* at a ratio of 1:20 (macrophage:bacteria) at 37 °C for various time periods. Bacterial uptake was assessed by FACScan analysis and bacterial ingestion was further determined after the external fluorescence of the bound, but non-ingested, bacteria was quenched with 0.025% crystal violet (Sigma-Aldrich). Intracellular bacterial killing was determined by incubation of macrophages with live *S. aureus* or *S. typhimurium* (macrophage:bacteria = 1:20) at 37 °C for various time periods in the presence or absence of cytochalasin B (5 μg/ml) (Sigma-Aldrich). After macrophages were lysed, total and extracellular bacterial killing were determined by incubation of serial 10-fold dilutions of the lysates on tryptone soy agar (Merck) plates at 37 °C for 24 hrs. Intracellular bacterial killing was calculated according to the total and extracellular bacterial killing.

### Measurement of phagosomal pH

Phagosome luminal pH was assessed as described previously^47^,^48^. Briefly, heat-killed *S. aureus* and *S. typhimurium* were doubly labelled with 5 μg/ml carboxyfluorescein-SE (a pH-sensitive fluorescent probe) (Molecular Probes, Eugene, OR, USA) and 10 μg/ml carboxytetramethylrhodamine-SE (a pH-insensitive fluorescent probe) (Molecular Probes). Isolated peritoneal macrophages were pulsed with the labelled bacteria (macrophage:bacteria = 1:20) for 20 min, and then chased at 37 °C for the indicated time periods. Macrophage-based mean fluorescence intensity (MFI) of fluorescein on FL1 and rhodamine on FL2 were simultaneously detected by FACScan analysis using CellQuest software (BD Biosciences, San Jose, CA, USA). Phagosomal pH was calculated according to the ratio of fluorescein:rhodamine fluorescence using a calibration curve.

### Assessment of phagosome maturation in a cell-free organelle system

Isolated peritoneal macrophages were labelled with a red fluorescent cell membrane linker PKH26 (20 μM) (Sigma-Aldrich) for subsequent phagosome recognition, as described previously^47^,^49^. PKH26-labelled macrophages were pulsed and chased with heat-killed *S. aureus* or *S. typhimurium* (macrophage:bacteria = 1:20) at 37 °C for the indicated time periods. Cells were lysed in a hypotonic buffer, and phagosomes were prepared by centrifugation. The isolated phagosomes were permeabilised with 0.2% saponin (Sigma-Aldrich) and stained with FITC-conjugated anti-LAMP-1 mAb (Abcam) that specially recognizes late endosomes/lysosomes, or FITC-conjugated isotype-matched mAb (Abcam) as the negative control. The green MFI of LAMP-1 on the positive red fluorescent events (phagosomes that have ingested bacteria), representing phagolysosome fusion and/or phagosome maturation, were quantitatively assessed by FACScan analysis using CellQuest software (BD Biosciences).

### Phagosome/lysosome fusion assay

Isolated peritoneal macrophages were plated into 8-well chamber slides (Lab-Tek™, Nunc, Rochester, NY, USA) at 1 × 10^5^ cells/well. After rested in RPMI1640 containing 1% FCS for 6 hrs, cells were loaded with LysoTracker red (50 nM) (Molecular Probes) at 37 °C for 30 min, and further incubated with *S. aureus* or *E. coli* conjugated with FITC (Molecular Probes) (macrophage:bacteria = 1:20) for various time periods. LysoTracker red was replenished every hour of incubation. After each time point, slides were vigorously washed 5 times in cold PBS and fixed in 2% paraformaldehyde (Sigma-Aldrich). Cell nuclei were stained with DAPI (Molecular Probes). Slides were mounted with coverslips and examined under a fluorescent Olympus BX61-TRF microscope (Olympus, Tokyo, Japan). Fluorescent images were acquired using the cell imaging software for life sciences microscopy (Olympus soft imaging solutions, Munster, Germany). Unfused phagosomes containing FITC-bacteria and lysosomes labelled with LysoTracker red were stained in green and red, respectively, whereas phagosomes containing FITC-bacteria after fused with LysoTracker red-labelled lysosomes were stained in yellow due to the coexistence of the two fluorochromes.

### Small interfering RNA transfection

Naive and BLP-tolerised BMMs were transfected with NOD1 and NOD2 specific siRNA or Rab10 specific siRNA (siRab10-1 and siRab10-2) by electroporation using a NEPA21 super electroporator (Nepagene, Chiba, Japan), and their scrRNA were used as the non-silencing control. Total RNA was extracted 24 hrs after transfection and the efficiency of interference was analyzed by quantitative real-time RT-PCR.

### RT^2^ profiler PCR arrays

RT^2^ profiler PCR arrays (Qiagen, Valencia, CA, USA) were used to profile gene expression of phagosome maturation-associated membrane-trafficking regulators and lysosomal enzymes. Total RNA was extracted from naïve and BLP-tolerised BMMs using an RNeasy kit (Qiagen) and the single strand cDNA from 2–3 μg total RNA was synthesised using an RT^2^ first strand kit (Qiagen). Real-time PCR was performed according to the user manual of RT^2^ profiler PCR array system. Thermal cycling and fluorescent detection were performed on the ABI 7900HT 384-well block detection system (Applied Biosystems, Foster City, CA, USA). All data were analysed using Excel-based RT^2^ profiler PCR array analysis templates (Qiagen).

### Quantitative real-time RT-PCR

Total RNA was extracted from naive and BLP-tolerised BMMs at the indicated time periods using TRIzol (Invitrogen) and reverse-transcribed into cDNA using the ReverTra Ace qPCR RT kit (Toyobo, Osaka, Japan). Amplification of cDNA was conducted using an ABI 7500 thermal cycler (Thermo Fisher Scientific, Waltham, MA, USA). The target gene mRNA expression was analyzed with FastStart universal SYBR green master (Roche Life Science, Indianapolis, IN, USA) and normalised with the housekeeping gene β-actin. The gene-specific primers used in this study were listed in Table 1.

### Western blot analysis

Following incubation with heat-killed *S. aureus* or *S. typhimurium*, naive and BLP-tolerised BMMs were collected at the indicated time periods, washed with ice-cold PBS, and lysed on ice in cell lysis buffer (Cell Signalling Technology), supplemented with 1 mM phenylmethylsulfonyl fluoride and protease inhibitor cocktail (Roche Life Science). The resultant lysates were centrifuged and supernatants containing the cytoplasmic proteins were collected. Protein concentration was determined using a micro BCA protein assay (Pierce, Rockford, IL, USA). Equal amounts of protein extracts were separated on SDS-polyacrylamide gels and transferred to polyvinylidene difluoride (PVDF) membranes (Schleicher & Schuell, Dassel, Germany). The membrane was blocked for 1 hr at room temperature with PBS containing 0.05% Tween-20 and 5% nonfat milk, and probed overnight at 4 °C with primary Abs. Blots were then incubated with appropriate horseradish peroxidase-conjugated secondary Abs (Dako, Cambridge, U.K.) at room temperature for 1 hr, developed with SuperSignal chemiluminescent substrate (Pierce), and captured with LAS-3000 imaging system (Fujifilm, Tokyo, Japan).

### Immunocytochemistry

Naive and BLP-tolerised BMMs were plated into 24-well chamber slides (Lab-Tek™) at 1 × 10^5^ cells/well. After rested in RPMI1640 containing 1% FCS for 6 hrs, cells were incubated with heat-killed *S. aureus* or *S. typhimurium* (macrophage:bacteria = 1:20) for the indicated time periods. After each time point, cells were washed 5 times in cold PBS, fixed in 2% paraformaldehyde, and permeabilised in 0.1% Triton X-100 for 10 min and 0.1% NaBH_4_ for 5 min. After blocked with PBS containing 0.05% Tween-20 and 3% BSA for 2 hrs, cells were incubated with the primary anti-p65 Ab overnight at 4 °C and further incubated with the Alexa Flour 594-conjugated secondary Ab (Molecular Probes) at room temperature for 1 hr. Cell nuclei were stained with DAPI (Molecular Probes). Slides were mounted with coverslips and images were taken under a LSM710 confocal laser scanning microscope (Zeiss, Oberkochen, Germany). The mean fluorescent intensity (MFI) of NF-κB p65 nuclear translocation was quantitatively measured as described previously^50^. Briefly, randomised 30 cells from at least five separate high-power fields per staining were selected for analysis. Image analysis of nuclear and cytoplasmic p65 fluorescent intensities was performed using the ImageJ software. The MFI of p65 nuclear translocation was calculated according to the ratio of nuclear to cytoplasmic p65 fluorescent intensities.

### Statistical analysis

All data are expressed as the mean ± SD. Statistical analysis was performed using Student’s t test or ANOVA, with GraphPad software version 5.01 (Prism, La Jolla, CA, USA). Differences were judged to be statistically significant when the *p* value was less than 0.05.

## Additional Information

**How to cite this article**: Liu, J. *et al*. NF-κB activation is critical for bacterial lipoprotein tolerance-enhanced bactericidal activity in macrophages during microbial infection. *Sci. Rep.* **7**, 40418; doi: 10.1038/srep40418 (2017).

**Publisher's note:** Springer Nature remains neutral with regard to jurisdictional claims in published maps and institutional affiliations.

## Supplementary Material

Supplementary Information

## Figures and Tables

**Table 1:** Gene-specific primers for quantitative real-time RT-PCR.

	Sense	Antisense
Acp2	5′-CTCTTCTCCAGTTCCTTCTTG-3′	5′-GTCCTTGGGATATGTCTTCAC-3′
Acp5	5′-CTTGCGACCATTGTTAGC-3′	5′-TTCTCGTCCTGAAGATACTG-3′
Rab10	5′-TGGAACTACAAGGAAAGAAGAT-3′	5′-TAGTAGGAGGTTGTGATGGT-3′
Rab20	5′-CCTTCTACCTGAAGCAGTG-3′	5′- TCGTATGTAAGGATGATAGCG-3′
Rabgef1	5′-GAGACTACTGATGATGAGAAGAA-3′	5′-GAGCATCTGAGGCGTTAC-3′
Camp	5′-CCAATCTCTACCGTCTCCT-3′	5′-GCCACATACAGTCTCCTTC-3′
Ctsc	5′-ACATTAACTGCTCGGTGAT-3′	5′-GTAGTCATTCAACACAATCTCG-3′
Nod1	5′-CTCCTCAGGAAGTTCGTTCG-3′	5′-ACCAGGTCAAGGATCTTTCG-3′
Nod2	5′-CCAACATTCGGAACACTCAG-3′	5′-GTACATGTCCGTGCTGGTTG-3′
β-actin	5′-GCGAGCACAGCTTCTTTGC-3′	5′-GCGCAGCGATATCGTCATC-3′
