# Supplementary material for: NF-κB activation is critical for bacterial lipoprotein tolerance-enhanced bactericidal activity in macrophages during microbial infection
Source: Sci Rep. 2017 Jan 12;7:40418. doi: 10.1038/srep40418 (PMC5227741; doi:10.1038/srep40418)
Supplement: Supplementary Information [file srep40418-s1.pdf]

NF-κB activation is critical for bacterial lipoprotein tolerance-enhanced bactericidal activity in macrophages during microbial infection

Jinghua Liu, Jing Xiang, Xue Li, Siobhan Blankson, Shuqi Zhao, Junwei Cai, Yong Jiang, H. Paul Redmond, and Jiang Huai Wang

Supplemetary Table 1. Gene expression of membrane-trafficking regulators and lysosomal enzymes in BLP-tolerised bone Marrow-derived macrophages (BMMs) by RT2 profiler PCR arrays

Refseq	Gene Symbol	Official Full Name	Fold Change	Trend
NM_007387	Acp2	Acid phosphatase 2, lysosomal	2.62	Up
NM_007388	Acp5	Acid phosphatase 2, tartrate resistant	15.71	Up
NM_008906	Ctsa	Cathepsin A	1.03	
NM_007798	Ctsb	Cathepsin B	0.49	Down
NM_009982	Ctsc	Cathepsin C	2.08	Up
NM_009983	Ctsd	Cathepsin D	0.44	Down
NM_007799	Ctse	Cathepsin E	0.01	Down
NM_019861	Ctsf	Cathepsin F	0.54	
NM_013590	Lyzl	Lysozyme 1	1.63	
NM_016722	Galns	Galactosamine (N-acetyl)-6-sulfate sulfatase	1.40	
NM_029364	Gns	Glucosamine (N-acetyl)-6-sulfatase	1.70	
NM_010498	Ids	Iduronate 2-sulfatase	1.41	
NM_018822	Sgsh	N-sulfoglucosamine sulfohydrolase (sulfamidase)	0.67	
NM_011421	Smpd1	Sphingomyelin phosphodiesterase 1, acid lysosomal	0.95	
NM_019734	Asah1	N-acylsphingosine amidohydrolase 1	0.94	
NM_001005847	Aga	Aspartylglucosaminidase	1.13	
NM_019441	Ppt2	Palmitoyl-protein thioesterase 2	1.23	
NM_021460	Lipa	Lysosomal acid lipase A	0.34	Down
NM_145937	Sumf1	Sulfatase modifying factor 1	1.32	
NM_021460	Hexa	Hexosaminidase A	0.64	
NM_010422	Hexb	Hexosaminidase B	0.47	Down
NM_008548	Man1a	Mannosidase 1, alpha	1.05	
NM_010764	Man2b1	Mannosidase 2, alpha B1	0.22	Down
NM_008079	Galc	Galactosylceramidase	0.54	
NM_008094	Gba	Glucosidase, beta, acid	1.99	
NM_008064	Gaa	Glucosidase, alpha, acid	1.23	
NM_008325	Idua	Iduronidase, alpha-L	0.93	

NM_017372	Lyz2	Lysozyme 2	1.64	
NM_028243	Prcp	Prolylcarboxypeptidase (angiotensinase C)	0.95	
NM_011189	Psme1	Proteasome (prosome, macropain) 28 subunit, alpha	0.78	
NM_009921	Camp	Cathelicidin antimicrobial peptide	15.58	Up
NM_010824	Mpo	Myeloperoxidase	0.65	
NM_019972	Sort1	Sortilin 1	0.73	
NM_019441	Ppt2	Palmitoyl-protein thioesterase 2	1.28	
NM_181414	Pik3c3	Phosphoinositide-3-kinase, class 3	0.82	
NM_025887	Rab5a	RAB5A, member RAS oncogene family	0.90	
NM_177411	Rab5b	RAB5B, member RAS oncogene family	0.90	
NM_009005	Rab7	RAB7, member RAS oncogene family	1.91	
NM_016676	Rab10	RAB10, member RAS oncogene family	2.52	Up
NM_024448	Rab12	RAB12, member RAS oncogene family	1.02	
NM_011227	Rab20	RAB20, member RAS oncogene family	2.16	Up
NM_033475	Rab34	RAB34, member of RAS oncogene family	0.72	
NM_001001932	Eea1	Early endosome antigen 1	0.88	
NM_010684	Lamp1	Lysosomal-associated membrane protein 1	1.19	
NM_010685	Lamp2	Lysosomal-associated membrane protein 2	0.99	
NM_007653	Cd63	Cd63 antigen	0.64	
NM_001029938	Rilp	Rab interacting lysosomal protein	0.64	
NM_053177	Mcoln1	Mucolipin 1	1.49	
NM_019400	Rabep1	Rabaptin, RAB GTPase binding effector protein 1	1.00	
NM_019983	Rabgef1	RAB guanine nucleotide exchange factor (GEF) 1	2.18	Up
NM_007508	Atp6v1a	ATPase, H+ transporting, lysosomal V1 subunit A	1.02	
NM_011596	Atp6vOa2	ATPase, H+ transporting, lysosomal V0 subunit A2	1.24	
NM_021433	Stx6	Syntaxin 6	0.92	
NM_016797	Stx7	Syntaxin 7	1.31	
NM_009295	Stxbp1	Syntaxin binding protein 1	1.04	
NM_177620	Rin3	Ras and Rab interactor 3	1.66	

The refseq, gene symbol, and the official full names are indicated. The fold change represents the ratio of the gene expression level in BLP-tolerised BMMs to that in naive BMMs. When the fold change is >2, indicating the upregulated (Up) gene expression, and when the fold change is <0.5, indicating the downregulated (Down) gene expression.

Figure S1

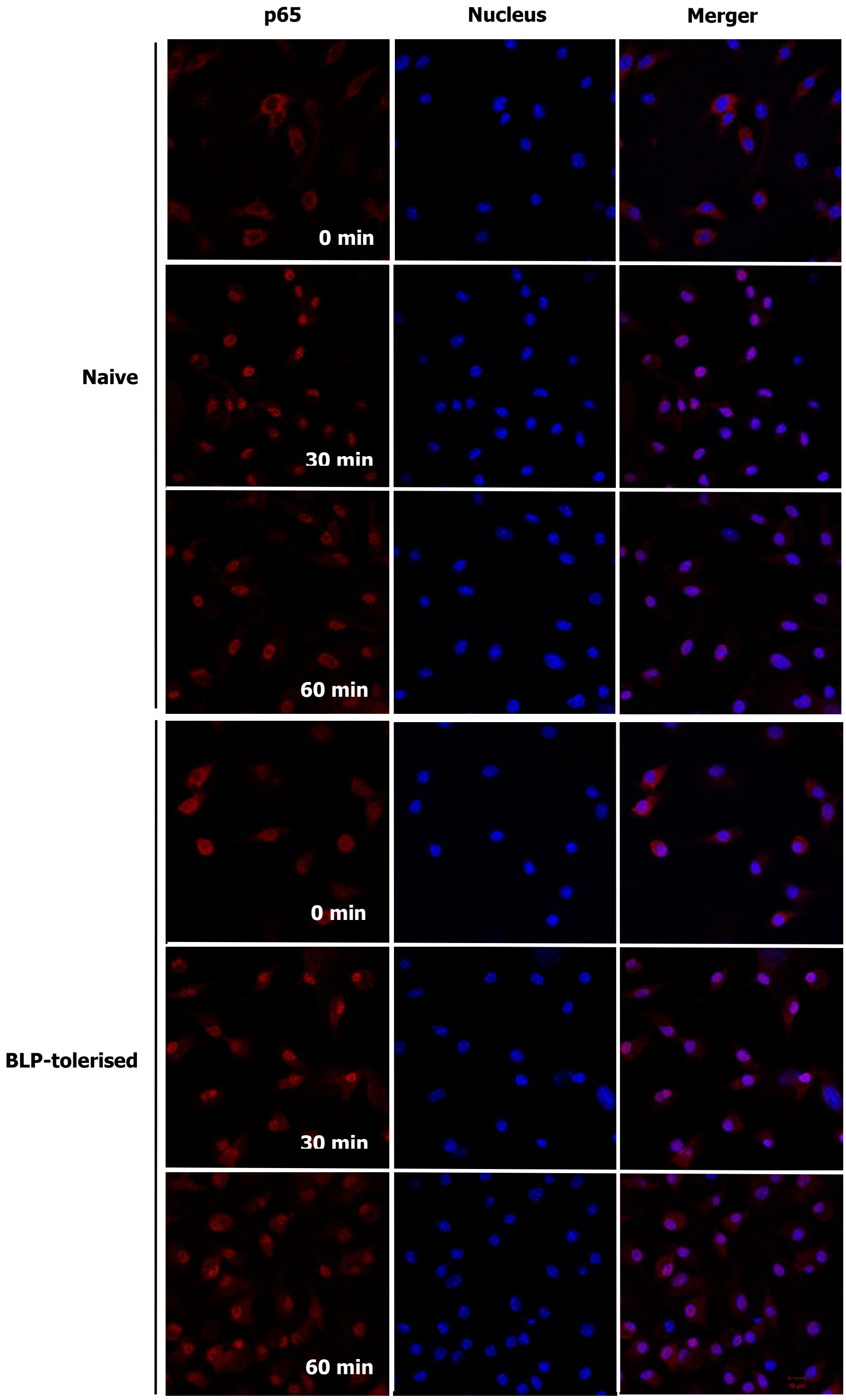

Fig. S1. Nuclear translocation of NF-κB p65 upon *S. typhimurium* infection in naive and BLP-tolerised macrophages. Naive and BLP-tolerised BMMs were stimulated with *S. typhimurium* for the indicated time periods. Confocal images were taken after cells were stained with the anti-p65 Ab and Alexa Flour 594-conjugated secondary Ab.

Figure S2

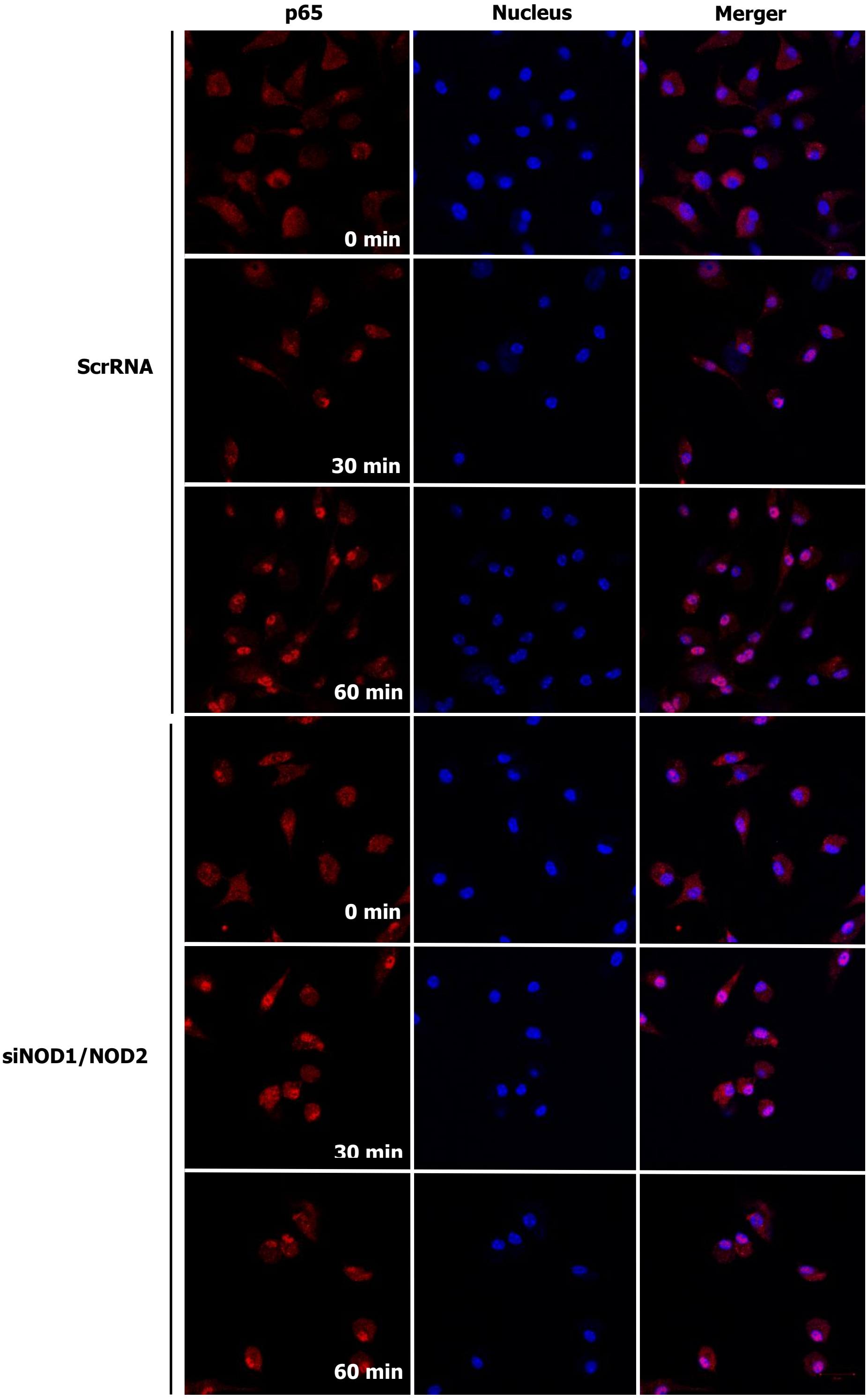

Fig. S2. Nuclear translocation of NF-κB p65 upon *S. typhimurium* infection in BLP-tolerised macrophages transfected with siNOD1/NOD2 or scrRNA. BLP-tolerised BMMs transfected with siNOD1/NOD2 or scrRNA were stimulated with *S. typhimurium* for the indicated time periods. Confocal images were taken after cells were stained with the anti-p65 Ab and Alexa Flour 594-conjugated secondary Ab.
